# Integrating artificial intelligence in osteosarcoma prognosis: the prognostic significance of SERPINE2 and CPT1B biomarkers

**DOI:** 10.1038/s41598-024-54222-6

**Published:** 2024-02-21

**Authors:** Haishun Qu, Jie Jiang, Xinli Zhan, Yunxiao Liang, Quan Guo, Peifeng Liu, Ling Lu, Yanwei Yang, Weicheng Xu, Yitian Zhang, Shaohang Lan, Zeshan Chen, Yuanhong Lu, Yufu Ou, Yijue Qin

**Affiliations:** 1 Guangxi Academy of Medical Sciences, People’s Hospital of Guangxi Zhuang Autonomous Region, Nanning, China; 2 Guangxi Hospital Division of The First Affiliated Hospital, Sun Yat-Sen University, Nanning, China; 3 Department of Spine and Orthopedic Surgery, The First Affiliated Hospital of Guangxi Medical University, Nanning, China

**Keywords:** Osteosarcoma, Artificial intelligence, Immune cell dysregulation, Prognostic model, Immunohistochemistry, Bone cancer, Cancer genomics, Oncogenes, Tumour biomarkers

## Abstract

The principal aim of this investigation is to identify pivotal biomarkers linked to the prognosis of osteosarcoma (OS) through the application of artificial intelligence (AI), with an ultimate goal to enhance prognostic prediction. Expression profiles from 88 OS cases and 396 normal samples were procured from accessible public databases. Prognostic models were established using univariate COX regression analysis and an array of AI methodologies including the XGB method, RF method, GLM method, SVM method, and LASSO regression analysis. Multivariate COX regression analysis was also employed. Immune cell variations in OS were examined using the CIBERSORT software, and a differential analysis was conducted. Routine blood data from 20,679 normal samples and 437 OS cases were analyzed to validate lymphocyte disparity. Histological assessments of the study's postulates were performed through immunohistochemistry and hematoxylin and eosin (HE) staining. AI facilitated the identification of differentially expressed genes, which were utilized to construct a prognostic model. This model discerned that the survival rate in the high-risk category was significantly inferior compared to the low-risk cohort (*p* < 0.05). SERPINE2 was found to be positively associated with memory B cells, while CPT1B correlated positively with CD8 T cells. Immunohistochemical assessments indicated that SERPINE2 was more prominently expressed in OS tissues relative to adjacent non-tumorous tissues. Conversely, CPT1B expression was elevated in the adjacent non-tumorous tissues compared to OS tissues. Lymphocyte counts from routine blood evaluations exhibited marked differences between normal and OS groups (*p* < 0.001). The study highlights SERPINE2 and CPT1B as crucial biomarkers for OS prognosis and suggests that dysregulation of lymphocytes plays a significant role in OS pathogenesis. Both SERPINE2 and CPT1B have potential utility as prognostic biomarkers for OS.

## Introduction

Osteosarcoma (OS) is the most prevalent primary malignancy originating from bone and soft tissues in children and adolescents and ranks third in adults^1^. The incidence of OS shows variations influenced by ethnicity and sex, with higher rates observed in African Americans compared to other ethnic groups in the United States, pointing towards a genetic predisposition^2^. The current therapeutic strategies for OS combine surgery with chemotherapy, which are often inadequate for this aggressive neoplasm^3^. As a highly malignant tumor, OS not only imposes substantial financial and psychological strains on patients and their families but also presents a critical threat to patient survival, underscoring the imperative to elucidate its pathogenesis.

Apoptosis, a form of programmed cell death, can be triggered by DNA damage and immune system activities. The interplay between apoptotic and other signaling pathways has been documented to culminate in cell death^4^. Notably, cancer cells often exhibit aberrant apoptotic processes, contributing to oncogenesis and presenting a challenge to effective tumor therapy due to resultant treatment resistance^5^. The complexity of the apoptotic mechanism, involving multiple pathways, suggests that any disruption may allow uncontrolled proliferation of malignant cells, leading to cancer development^6^. In this study, we sought to identify biomarkers correlated with OS prognosis by analyzing apoptosis-related gene sets from the Gene Set Enrichment Analysis (GSEA) database to assess the role of apoptosis in OS.

Artificial intelligence (AI) is revolutionizing various domains, including medicine, enhancing both physician and patient experiences and promising more accurate, convenient, and efficacious medical interventions globally^7^. AI's burgeoning application in early disease diagnosis and prognosis is poised to augment clinical decision-making and healthcare outcomes^8^. Recent literature underscores AI's potential in identifying disease biomarkers and prognostic indicators that guide clinical practices^9–11^. However, the integration of AI and apoptotic mechanisms in the context of OS remains underexplored, which our study aims to address by utilizing AI to identify pivotal genes and constructing prognostic models for OS.

In recent years, AI has demonstrated its potential across multiple medical fields, particularly in the prognosis of diseases and in making treatment decisions. In the study of Overall Survival (OS) prognosis, AI technology has shown unique advantages in analyzing big data and pattern recognition^12^. These technologies can not only handle a vast amount of clinical data but also identify correlations between complex biomarkers and patient characteristics, thereby enhancing the accuracy of prognoses^13^. Specifically in predicting OS, AI methods such as machine learning and deep learning have been proven effective in analyzing patients' survival data, including genomic information, clinical features, and treatment responses. These methods can identify key prognostic indicators within complex datasets, thereby assisting physicians in developing more personalized treatment plans. Through precise OS predictions, medical teams can better understand disease progression, offering targeted treatment and care strategies for patients.

The tumor microenvironment (TME) has become a focal point in cancer research. Recent findings indicate that targeting TME components with specific drugs may offer new avenues for treating metastatic and progressive ovarian cancer^14^. Cancer therapies typically function by eliciting immune responses or directly inducing tumor cell death^15^. Furthermore, a study has demonstrated the efficacy of an RNA-LPX vaccine delivered intravenously as immunotherapy in melanoma patients resistant to checkpoint inhibitors^16^, emphasizing the significance of immunotherapy in cancer treatment. Xu et al. presented that CAR T-cell therapy's effectiveness against solid tumors is hindered by the challenges of persistence and TME infiltration. They found, through single-cell RNA sequencing, that DMXAA can enhance CAR T-cell trafficking and persistence within a chemokine-rich environment, which modulates and augments CAR T-cell recruitment by balancing stimulatory and suppressive TME dynamics^17^. Interestingly, advanced cutaneous T-cell lymphoma patients exhibit immune dysfunction, are prone to infections, and have impaired tumor immune responses^18^, suggesting the relevance of immune cells in oncology.

This study is dedicated to developing a prognostic model for OS employing sophisticated bioinformatics tools, investigating the correlation between the selected genes and immune cells, and validating these associations with immunohistochemical staining. The primary goal is to investigate the potential mechanisms by which dysregulated genes and immune cells contribute to the progression of OS.

## Materials and methods

### Acquisition of osteosarcoma expression profiles, normal controls, and apoptosis-related gene data

OS Expression Profiles: The specific expression profiles for OS were retrieved from the University of California Santa Cruz Xena platform, a robust database amalgamating genomic and clinical data. The accessibility of this database simplifies the process for researchers to obtain necessary data efficiently.

Control Group Expression Profiles: In order to establish a baseline for comparison, expression data for normal tissues were acquired from the Genotype-Tissue Expression (GTEx) project database. Here, skeletal muscle tissue expression profiles were specifically chosen to serve as the control group, representing the normal counterpart to the OS samples. We employed both oversampling and undersampling techniques to balance our dataset. Oversampling was achieved by replicating samples from the minority class, while undersampling involved reducing the number of samples in the majority class.

Normalization and Log Transformation: Following download, the raw data underwent a standardization procedure involving normalization and log2 transformation. These steps are critical for reducing variability and enhancing the comparability of gene expression levels across different samples. The R programming environment (version R × 64 4.0.2) was the chosen tool for executing these data processing tasks, given its comprehensive suite of statistical functions and packages tailored for genomic data analysis.

Apoptosis-Related Genes: The Gene Set Enrichment Analysis (GSEA) database was utilized to obtain a curated list of genes associated with the apoptotic process. Apoptosis is a form of programmed cell death, pivotal to understanding cancer biology and potential therapeutic intervention points.

**Figure 1:**
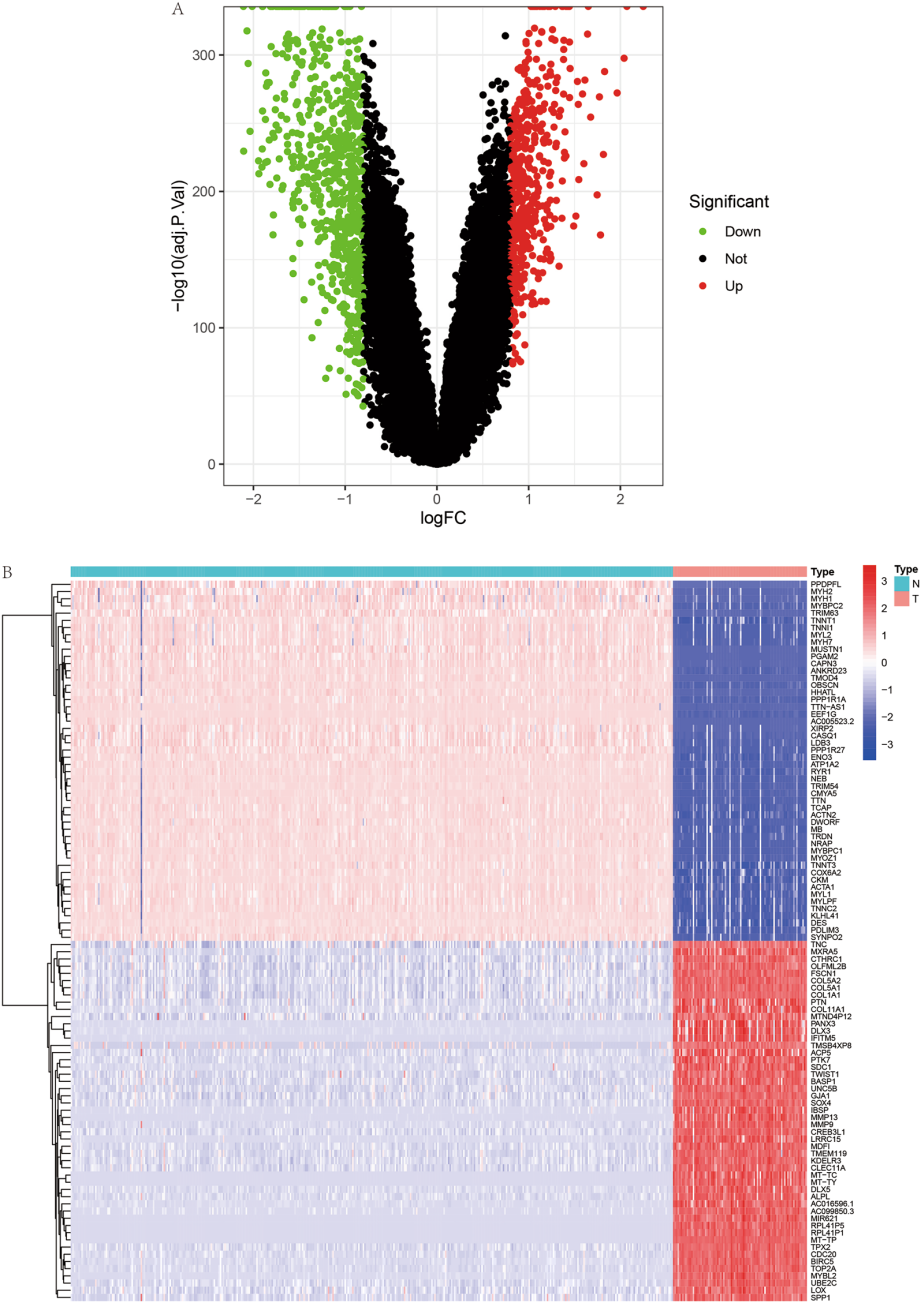
Differential gene expression profiles. (**A**) The volcano plot (A) displays differentially expressed genes, with upregulated genes highlighted in red and downregulated genes in green. (**B**) The heatmap (B) illustrates the differential expression patterns, where red signifies high expression and blue indicates low expression.

Validation Dataset: To solidify the findings and ensure their applicability, the GSE21257 dataset from the Gene Expression Omnibus (GEO) was employed as a validation set. This dataset encompasses gene expression profiles from 53 OS samples and serves as a crucial resource for verifying the prognostic or diagnostic relevance of identified gene expression patterns in OS. We have placed the workflow diagram in Fig. 1.

### Differential expression analysis

Following the identification of differentially expressed genes (DEGs), the data were visualized using two types of graphical representations. The first, volcano plots, provided a powerful visual framework to display the vast landscape of gene expression changes, highlighting those with both substantial fold changes and high statistical significance. The second, a heat map, offered an intuitive color-coded representation of the expression levels of the top 100 DEGs. This not only underscored the expression magnitude of these genes but also allowed for a clear comparison across samples, showcasing patterns and potential clusters of gene expression. These visual tools are crucial for a rapid and discernible assessment of the data, facilitating the identification of candidate genes for further investigation.

### Enrichment analysis of apoptosis-related genes

Exploring the intricate role of apoptosis in osteosarcoma (OS) necessitated the extraction of apoptosis-related genes from the Gene Set Enrichment Analysis (GSEA) database. A subsequent step was to delineate the subset of these genes that were differentially expressed in the context of OS, forming a focused group for further scrutiny. The analysis then proceeded with Gene Ontology (GO) enrichment and Kyoto Encyclopedia of Genes and Genomes (KEGG) pathway analyses. These analyses aimed to elucidate the roles and mechanistic pathways that the apoptosis-related differentially expressed genes may partake in, offering insights into their functional dynamics and potential influence on the pathophysiology of OS.

### Artificial intelligence screening for apoptosis-related genes

To refine the search for prognostic biomarkers in OS, this study employed a cadre of artificial intelligence (AI) techniques: Support Vector Machine (SVM), Random Forest (RF), Generalized Linear Model (GLM), and Extreme Gradient Boosting (XGBoost). These sophisticated machine learning methods were utilized to sieve through the pool of differentially expressed genes, with a particular focus on those implicated in apoptosis. The integration of these AI approaches aimed to augment the predictive accuracy and reliability of the biomarker discovery process. The final set of candidate biomarkers, distilled using these AI methods, was then visually represented, furnishing a comprehensive view of the potential prognostic markers for OS. During the training phase, each model—SVM, RF, GLM, and XGBoost—was independently trained on the training set. Model parameters were fine-tuned to optimize performance metrics such as accuracy, sensitivity, and specificity. In the testing phase, the performance of each model was evaluated on the held-out test set to assess its predictive power and generalizability. Furthermore, to prevent overfitting and ensure the robustness of our models, techniques such as grid search and regularization (specifically in the case of GLM and XGBoost) were employed.

### Construction of a prognostic model for osteosarcoma

To elucidate the prognostic significance of apoptosis-related differentially expressed genes in osteosarcoma (OS), an in-depth analysis led to the construction of a prognostic model. This model’s foundation lay in the rigorous screening of genes through a tri-phasic methodological approach, ensuring a robust selection process that mitigates multicollinearity. The preliminary phase of our analysis involved the application of univariate Cox regression analysis. This step was critical for the identification of candidate genes, with a significance threshold set at *p* < 0.05. For detailed results and a comprehensive list of these genes, refer to Table 1. Subsequently, the LASSO (Least Absolute Shrinkage and Selection Operator) regression honed the model, introducing penalty coefficients that trimmed the gene list to a core selection. We utilized the glmnet package in R to implement LASSO regression. For the selection of the regularization parameter (lambda) in our LASSO model, we employed a tenfold cross-validation approach. Specifically, we tested a range of lambda values to identify the one that minimizes the cross-validation error. The chosen lambda value ensures a balance between model complexity and predictive accuracy, achieving an optimal level of regularization. The final phase involved multivariate Cox regression analysis, reinforcing the association of these genes with OS outcomes at a significance level of *p* < 0.05. For detailed results and a comprehensive list of these genes, refer to Table 2. In this study, we have implemented a detailed model training and testing protocol within the framework of tenfold cross-validation to ensure minimal bias in model evaluation. Specifically, the dataset was first randomly partitioned into 10 equal-sized subsets. In each fold of the cross-validation, one subset was retained as the test set while the remaining nine subsets were amalgamated to form the training set. This process was iteratively repeated ten times, with each subset being used exactly once as the test set.

**Table 1:** Results of univariate Cox regression analysis. Table 1 shows the specifics of the univariate Cox regression analysis.

id	HR	HR.95L	HR.95H	pvalue
CADM1	1.532487	1.051916	2.232608	0.026175
CD24	1.268944	1.029597	1.563931	0.025518
CDH11	0.650804	0.436076	0.971267	0.035494
CDK1	1.752635	1.083551	2.834872	0.022194
GM2A	0.608848	0.375945	0.986038	0.043683
KCNMA1	1.459825	1.036846	2.055356	0.030216
RRAD	1.300187	1.030339	1.640708	0.026981
SERPINH1	1.943459	1.168231	3.233119	0.010506
MYOM2	1.48061	1.126312	1.946357	0.004918
SERPINE2	1.520079	1.18292	1.953337	0.001065
SQLE	1.698986	1.268753	2.275112	0.000374
ZC3HAV1	2.16794	1.219418	3.854268	0.008397
TYROBP	0.809892	0.65775	0.997225	0.047018
UNC5B	1.728302	1.157866	2.579772	0.007424
COL5A2	1.612495	1.139022	2.282782	0.007062
CPT1B	11.21139	1.485484	84.61575	0.019093
EIF4A1	1.880612	1.033776	3.421149	0.038568
HJURP	2.250387	1.244968	4.067769	0.007245
LOX	1.627401	1.200866	2.205437	0.001688
MYOC	45,722.53	3.050442	6.85E + 08	0.02872
NUF2	1.612883	1.039131	2.503429	0.033082
PAPSS1	0.566762	0.324739	0.989163	0.045683
PHKA1	1.585717	1.065551	2.35981	0.023027
SCD	1.356157	1.075024	1.710811	0.010161
SDC3	0.623488	0.449581	0.864667	0.004633
SLC29A2	1.70689	1.093235	2.665002	0.018667
ACAD11	904.5942	3.436298	238,131.5	0.016662
C19orf47	0.396313	0.18494	0.849272	0.017308
COL11A2	1.252309	1.080133	1.451929	0.002869
EIF4E3	0.33451	0.144418	0.774812	0.01061
FOLR1	1.346694	1.087184	1.668148	0.006424
PFKFB2	0.09036	0.019362	0.42169	0.002224
SGCA	1.25511	1.044198	1.508623	0.01549
COL4A3	8.880001	1.119117	70.46127	0.038785

**Table 2:** Results of multivariate Cox regression analysis. Table 2 shows the specifics of the multivariate Cox regression analysis.

id	coef	HR	HR.95L	HR.95H	pvalue
SERPINE2	0.4683	1.597277	1.23067	2.073093	0.000431
CPT1B	3.078747	21.73116	2.422481	194.942	0.005952

### Survival analysis of patients with osteosarcoma

Survival trajectories for OS patients were delineated through Kaplan–Meier curves, anchored to the expression levels of autophagy-related differentially expressed genes. Stratification of OS cases into high and low expression cohorts based on a median value criterion allowed for comparative survival analysis. Concurrently, an OS prognostic model was deployed, categorizing patients into risk-defined groups derived from a calculated risk score. Patients with scores surpassing the mean risk score fell into the high-risk category, whereas those at or below were deemed low-risk. The resultant Kaplan–Meier plots for these cohorts revealed significant survival disparities, notably illustrated in Fig. 5L, where the model, predicated on two pivotal genes within the GSE21257 dataset, underscored a stark survival disadvantage in the high-risk group (*p* < 0.001).

### Reliability test of the prognostic model

The prognostic model’s fidelity was scrutinized through a triad of evaluative techniques. ROC (Receiver Operating Characteristic) curve analysis initially provided a survival rate-based diagnostic check across 1-year, 3-year, and 5-year benchmarks. The comparison between high-risk and low-risk groups ensued, examining gene expression variations implicated in the model’s architecture. Additionally, calibration plots served to contrast predicted outcomes against actual data, offering a tangible measure of the model’s predictive accuracy. To cement the model's validity, it was cross-examined using the defining genes within the GSE21257 dataset, thus ensuring the model's reliability.

### Analysis of immune cell composition

In the quest to unravel the interplay between immune cell dynamics and osteosarcoma (OS), CIBERSORT software emerged as a pivotal analytical tool. This advanced software is specifically designed for deconstructing the composite expression matrices of immune cell subtypes, employing linear support vector regression as its computational backbone. With its distinctive algorithm, CIBERSORT precisely quantifies the constituent immune cells within the expression profiles of OS samples. This quantification is critical for establishing correlations between specific immune cell infiltrations and the gene signatures that were integral to the construction of the prognostic model. The insights garnered from this analysis could potentially illuminate the role of the immune microenvironment in OS pathogenesis and prognosis, providing a deeper understanding of the tumor-immune interplay at the molecular level.

### Immunohistochemistry analysis

Immunohistochemistry (IHC) was conducted to validate the findings of our analysis rigorously. Pathological tissue specimens utilized for the IHC staining were procured during surgical procedures at the First Clinical Affiliated Hospital of Guangxi Medical University. The study was conducted with the approval of the hospital’s ethics committee, in alignment with the ethical standards of the Declaration of Helsinki. Considering the anonymous nature of the patient tissue samples in this IHC study, a waiver for informed consent was sought.

Specific antibodies targeting SERPINE2 were acquired from Proteintech (Catalog number: 66203-1-Ig, available at Proteintech), and those for CPT1B were sourced from ABclonal (Item number: A6796, accessible at ABclonal). Tissue preparation involved prompt formalin fixation within 15 min of excision, followed by sequential processing including dehydration, wax infiltration, embedding, sectioning, deparaffinization, and rehydration. Antigen retrieval, endogenous peroxidase blocking, and incubation with primary and secondary antibodies were systematically performed. After the staining process, tissues were developed in chromogen and counterstained.

The stained slides were examined and imaged using an inverted microscope. This was followed by hematoxylin and eosin (H&E) staining to highlight cellular and tissue structures, encompassing the nuclei staining with hematoxylin and cytoplasmic staining with eosin. Finally, the slides were dehydrated, cleared, and mounted for detailed examination and imagery under an inverted microscope.

### Analysis of routine blood data to assess lymphocyte discrepancies

To corroborate the lymphocyte differentiation ascertained by the CIBERSORT software analysis, this investigation gathered a substantial dataset of routine blood examinations. For this study informed consent has been waived by The First Affiliated Hospital of Guangxi Medical University Institutional review board (IRB)/ethics committee. The control group was composed of non-osteosarcoma (non-OS) and non-tumor bearing individuals, using records from a decade-long period (January 1, 2012, to January 1, 2022) at the First Affiliated Hospital of Guangxi Medical University, reflecting a healthy baseline. In contrast, the experimental group consisted of patients with diagnosed OS, from which routine blood data was obtained. The two groups' lymphocyte counts were statistically examined using the t-test for differences in means.

R programming language was utilized for the statistical analysis and graphical visualization of the data, providing a clear comparative representation of the lymphocyte levels between the healthy control and OS patient groups. This comparative analysis aimed to validate the consistency of lymphocyte differences identified by the advanced computational method provided by CIBERSORT, thereby ensuring the robustness of the immunological insights derived from the study.

### Ethical approval

This study was approved by the Ethics Review Committee of the People's Hospital of Guangxi Zhuang Autonomous Region and was in accordance with the Declaration of Helsinki of the World Medical Association.

## Results

### Osteosarcoma gene expression and control data analysis

We sourced data for 88 osteosarcoma cases from the UCSC Xena platform and used 396 skeletal muscle samples from GTEx as a normal control set. They also compiled a list of 4675 apoptosis-related genes from the GSEA database for targeted investigation.

A comprehensive differential expression analysis across 54,751 genes was conducted, pinpointing 1,197 genes that differed significantly between osteosarcoma tissues and the control group. These genes were depicted in a volcano plot (Fig. 1A), where red dots signified genes with elevated expression levels and green dots represented genes with reduced expression in osteosarcoma. Additionally, a heat map (Fig. 1B) showcased the expression patterns of the top 100 significantly altered genes, with varying shades of red and blue indicating the levels of expression.

### Go and KEGG enrichment

**Figure 2:**
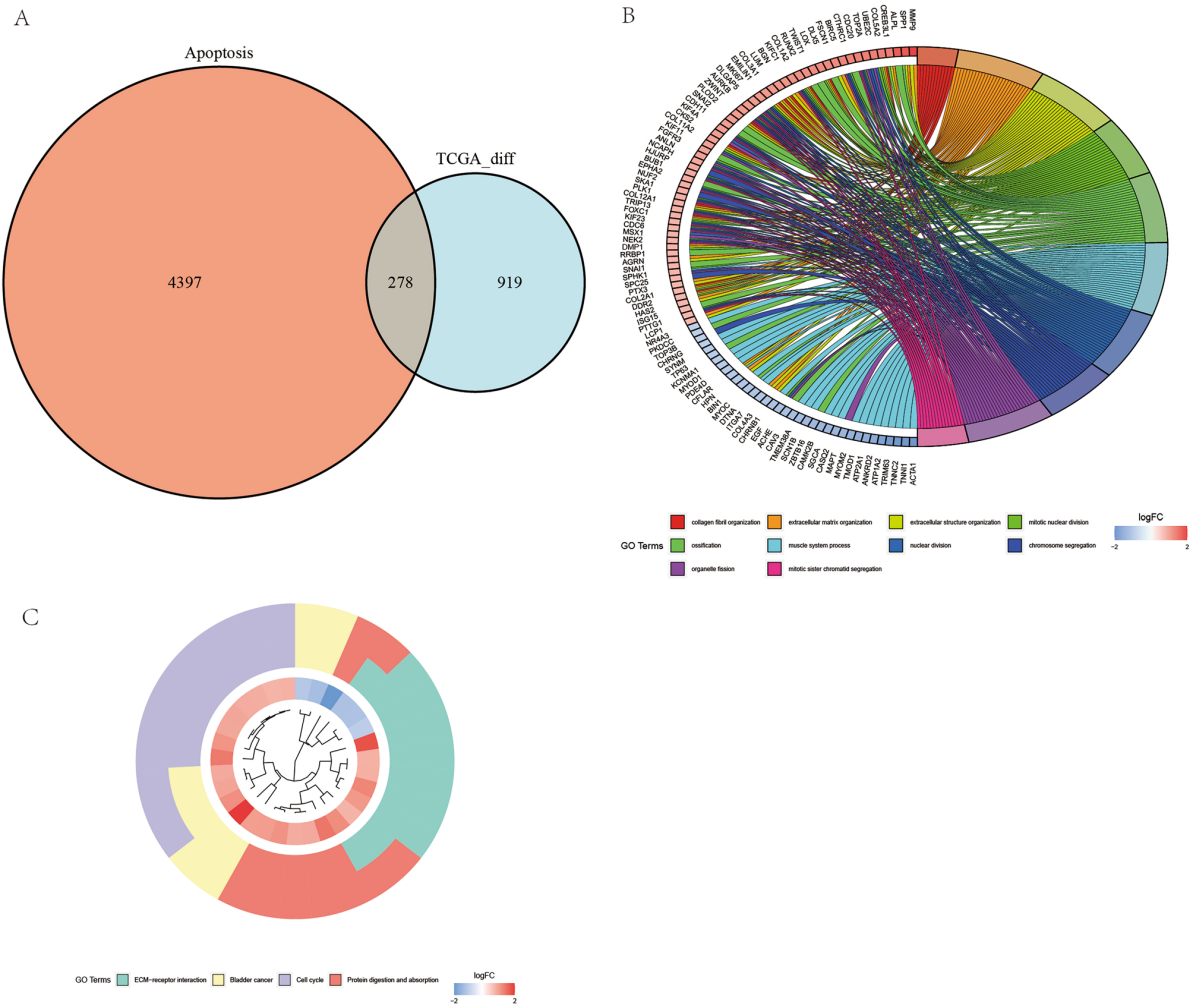
Intersection and enrichment analyses of apoptosis-related genes. (**A**) The Venn diagram (**A**) shows the overlap between apoptosis-associated genes and differentially expressed genes. (**B**) The bar graph (**B**) represents the Gene Ontology (GO) enrichment analysis outcomes for genes linked to apoptosis. (**C**) The bubble chart (**C**) displays the Kyoto Encyclopedia of Genes and Genomes (KEGG) pathway enrichment analysis for apoptosis-related genes.

From Fig. 2A, it is evident that there are 278 intersecting genes between genes related to apoptosis and differentially expressed genes.Gene Ontology (GO) Enrichment: The GO enrichment analysis, depicted in Fig. 2B, highlighted the top biological processes that these apoptosis-related genes are involved in. Key processes included: extracellular matrix organization Mitotic nuclear division and related processes like chromosome segregation, Bone formation (ossification), Muscle system processes. These biological processes underscore the intricate relationship between cellular structure, division, and movement, all of which are pivotal in both normal physiology and cancer pathology.

Kyoto Encyclopedia of Genes and Genomes (KEGG) Pathway Enrichment. In the KEGG pathway analysis (Fig. 2C), researchers discovered that the apoptosis-related genes were significantly enriched in pathways known to be integral to cancer biology, such as: ECM-receptor interaction, Cell cycle regulation, Specific cancer pathways, including bladder cancer and Protein digestion and absorption.This enrichment in both cellular component organization and cancer-specific pathways highlights the potential role of these apoptosis-related genes in the development and progression of OS. It also suggests that the extracellular matrix and cell cycle regulation are key areas of interest for understanding OS pathology and potential therapeutic targets.

### AI screening

**Figure 3:**
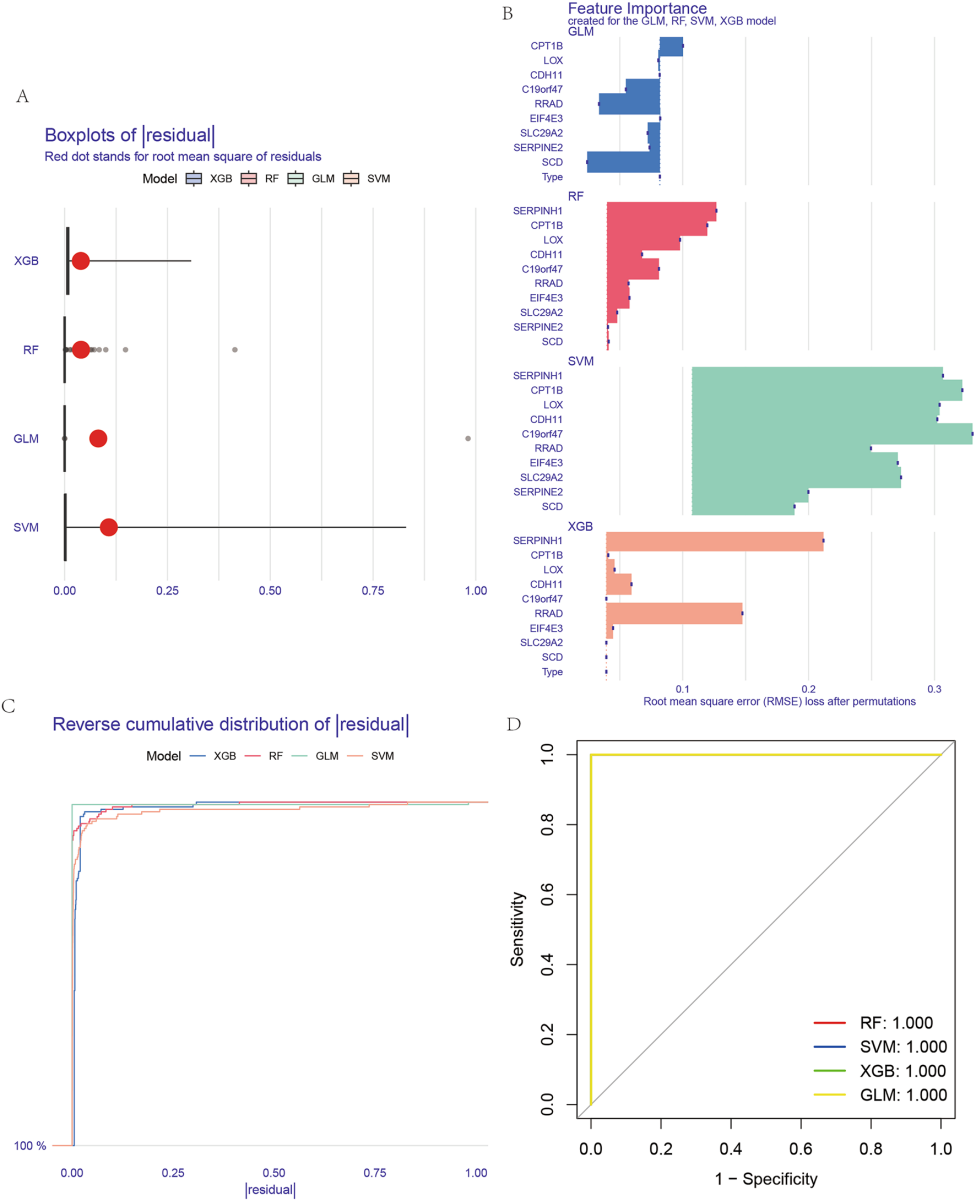
Overview of AI screening results. (**A**) The residual boxplot (**A**) compares the residuals across the four AI screening methods. (**B**) The intersection diagram (**B**) illustrates the genes identified after the individual AI screenings. (**C**) The reverse cumulative distribution plot (**C**) visualizes the distribution of residuals. (**D**) The ROC curves (**D**) display the diagnostic performance of the four AI screening techniques.

Using advanced artificial intelligence (AI) methodologies, the study delved into apoptosis-related differentially expressed genes with a high-throughput screening approach. The techniques employed included Support Vector Machine (SVM), Random Forest (RF), Generalized Linear Model (GLM), and Extreme Gradient Boosting (XGBoost). Model Residuals: In Fig. 3A, the residuals of the four AI models were depicted. Residuals represent the differences between observed and predicted values by the models, with smaller residuals suggesting a better fit to the data. Top Genes Identified: The ten most significant genes identified by each of the AI methods were listed in Fig. 3B. These genes are potential biomarkers for prognosis and may play a significant role in the apoptotic pathways within OS. Cumulative Distribution of Residuals: Fig. 3C illustrated a cumulative distribution function for the residuals of the models. A steep curve in this distribution would suggest a majority of predicted values are close to the actual data, signifying a high accuracy. ROC Diagnostic Curves: The Receiver Operating Characteristic (ROC) curves for each AI method were shown in Fig. 3D. These curves are tools used to assess the diagnostic performance of a binary classifier system. The closer the ROC curve is to the top left corner, the higher the accuracy of the test. In this case, the convergence of ROC curves towards 100% diagnostic efficacy indicates an exceptionally high predictive power of the AI methods in distinguishing between apoptosis-related differentially expressed genes and others. Overall, the application of these AI techniques provided a robust screening process for identifying key genes involved in apoptosis in OS, which might offer new insights into the pathogenesis of the disease and open avenues for developing targeted therapies.

### Development of an apoptosis-related gene prognostic model for osteosarcoma

**Figure 4:**
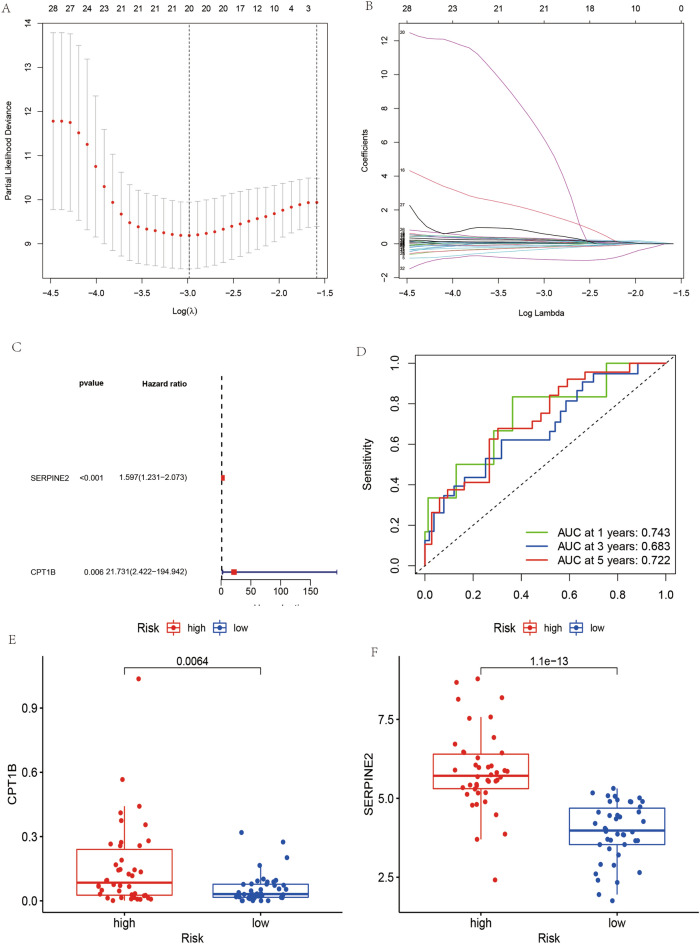
Prognostic model construction and analysis. (**A**) LASSO coefficient profiles of the prognostic genes, with plot A demonstrating the selection of the optimal parameter (lambda) in the LASSO model. (**B**) Validation of the LASSO model’s gene selection, showing the cross-validation curve. (**C**) Forest plot (**C**) indicating the hazard ratios and confidence intervals for the two prognostic genes utilized in the model construction. (**D**) ROC curve analysis (D) depicting the predictive accuracy of the constructed prognostic model. (**E**, **F**) Expression levels of the two prognostic genes in high-risk (**E**) and low-risk (**F**) groups, respectively, highlighting their differential expression and potential prognostic significance.

Univariate Cox regression analysis identified thirty-four genes significantly correlated with osteosarcoma (OS) prognosis (*P*-value < 0.05). LASSO regression refined the predictive model, pinpointing twenty genes of substantial prognostic relevance (refer to Fig. 4A,B). Subsequent multivariate Cox regression analysis delineated two genes, SERPINE2 and CPT1B, as robust prognostic markers (illustrated in Fig. 4C). The model's diagnostic precision was validated through receiver operating characteristic (ROC) curves, which demonstrated an area under the curve (AUC) substantially above 0.5 for predictions of 1-year, 3-year, and 5-year patient survival (depicted in Fig. 4D). In risk score assessment, patients were categorized into high and low-risk groups based on the median risk score calculated from the gene set. Elevated expression levels of CPT1B and SERPINE2 were associated with higher risk and reduced survival prospects (evident in Fig. 4E,F). The prognostic model's potent predictive capability underscores its potential application in the clinical prognostication of OS patient outcomes.

### Survival analysis of osteosarcoma patients

**Figure 5:**
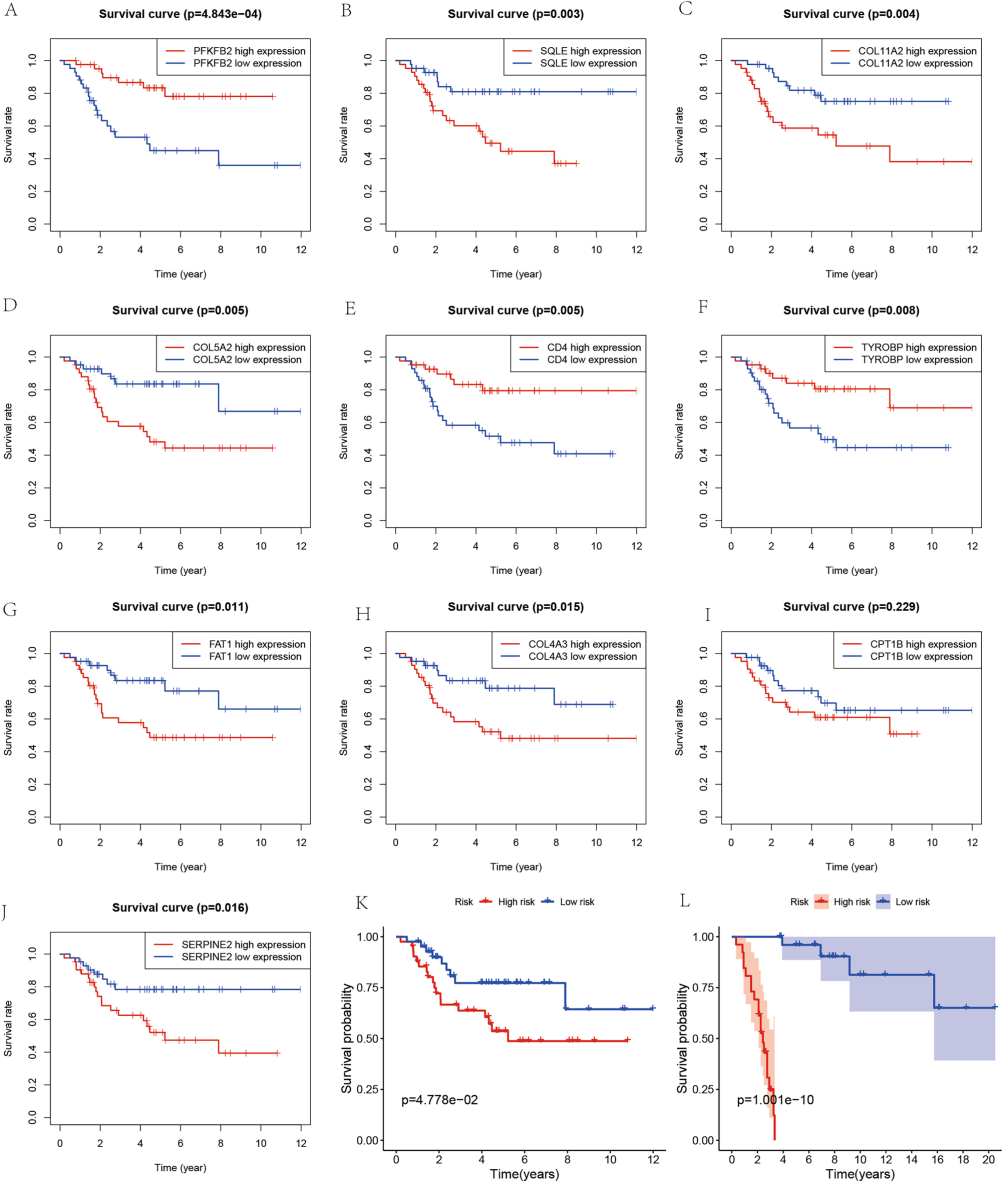
Survival Analysis and Prognostic Validation. (**A**–**J**) Kaplan–Meier survival curves for ten genes, comparing the overall survival of patients with high vs. low expression levels of each gene. Each plot (**A**–**J**) corresponds to a specific gene, illustrating its impact on patient survival. (**K**) Survival analysis based on the prognostic model, with the Kaplan–Meier curve contrasting the outcomes of high-risk versus low-risk groups as defined by the model, underscoring the model's predictive capacity for patient prognosis. (**L**) External validation of the prognostic model using the survival curve derived from dataset GSE21257. This plot (L) confirms the model's reliability and applicability to independent patient cohorts.

Survival analysis was conducted to evaluate the prognostic implications of gene expression levels in osteosarcoma (OS) cases. The study employed Kaplan–Meier survival curves (Fig. 5A–J) to assess the impact of ten apoptosis-related genes that exhibited significant differential expression. From these genes, SERPINE2 and CPT1B were particularly notable. The Kaplan–Meier plots revealed a statistically significant disparity in survival between high and low expressions of SERPINE2 (Fig. 5J), with elevated SERPINE2 levels correlating with poorer patient outcomes (*p* < 0.05). However, the survival differences between the high and low expression strata of CPT1B were not statistically significant (Fig. 5 I, *p* > 0.05).

The prognostic model developed, integrating these genes, showed a marked distinction in survival outcomes between the defined high-risk and low-risk groups (Fig. 5K), with the high-risk group demonstrating significantly lower survival rates (*p* < 0.05). Further validation of this prognostic model using the GSE21257 dataset confirmed its predictive value; individuals classified within the high-risk category according to the model had substantially reduced survival compared to those in the low-risk category (Fig. 5L). This evidence substantiates the model's potential as a predictive tool in the clinical management of osteosarcoma.

### Evaluation of the prognostic model for osteosarcoma

The prognostic model developed for osteosarcoma (OS) was subject to thorough validation to ascertain its precision. Diagnostic performance was initially assessed using receiver operating characteristic (ROC) curves (Fig. 4D), where the area under the curve (AUC) consistently exceeded 0.65 for predictions at 1, 3, and 5 years, indicating a high level of model accuracy.

Furthermore, expression levels of the two genes central to the model, CPT1B and SERPINE2, were compared between the high-risk and low-risk groups (Fig. 4E). In both instances, the high-risk group showed a statistically significant higher mean gene expression (*p* < 0.05), reinforcing the genes’ prognostic relevance.

**Figure 6:**
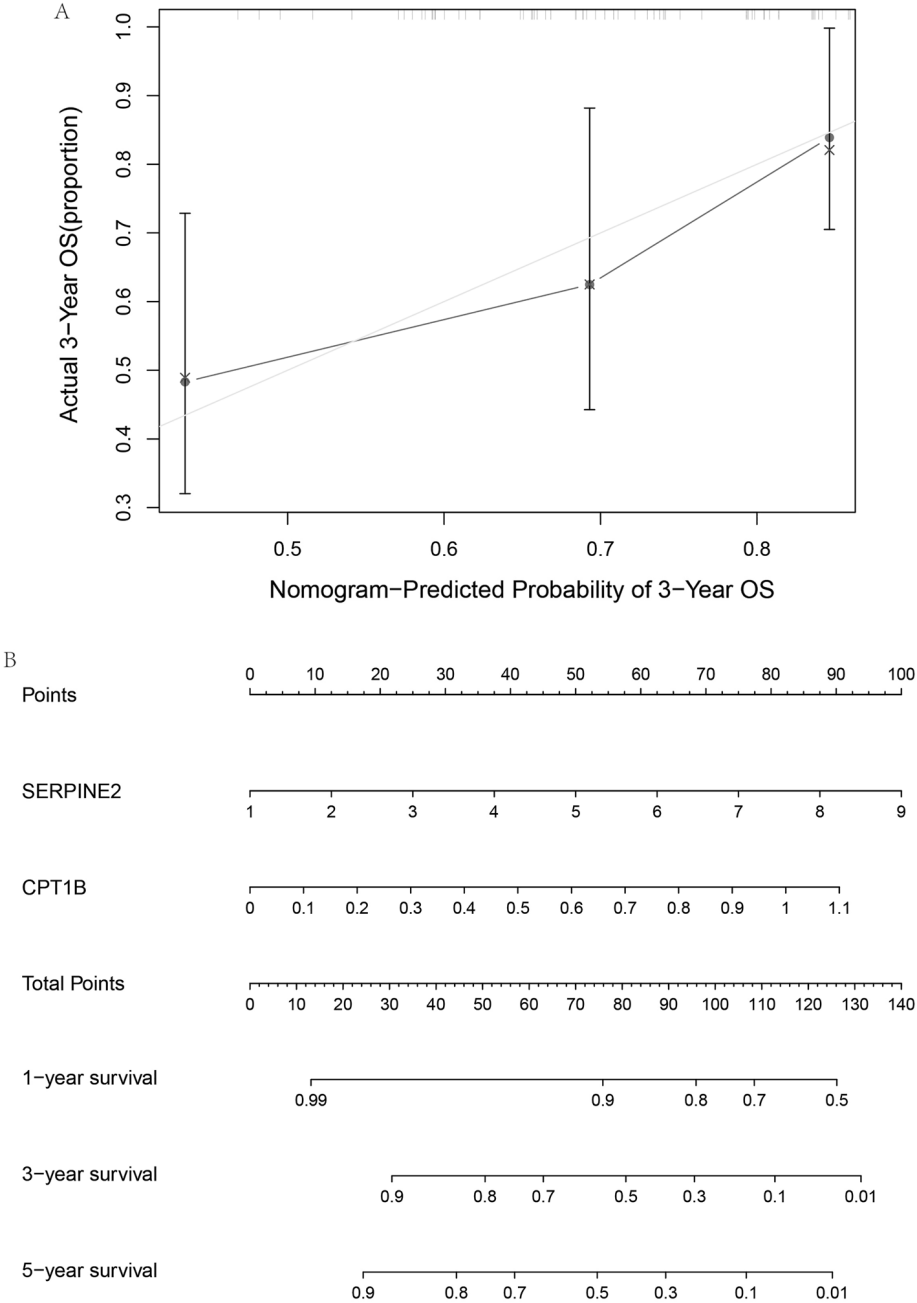
Prognostic Prediction Assessment. (**A**) The calibration plot for prognostic prediction demonstrates the accuracy of the prognostic model. The closer the curve is to the 45-degree line, the more the predicted survival probabilities align with the observed outcomes. (**B**) The column plot for predicted prognosis provides a visual comparison of the predicted versus actual survival status of patients. Each column represents an individual patient, with the height indicating the probability of survival as predicted by the model.

Complementing these findings, a calibration plot (Fig. 6A) was employed as a more stringent test of the model’s predictive capability. The plot demonstrated that the model's prediction for survival onset was slightly higher than the actual observed values, but by the endpoint, predicted and actual values were closely aligned, confirming the model's accuracy over time.

Lastly, the expression of SERPINE2 and CPT1B was presented using columnar plots (Fig. 6B) to visually correlate gene expression with patient survival rates. The ability to predict OS patient outcomes based on the expression of these genes highlights the clinical potential of the model in guiding prognosis and treatment strategies.

### Analysis of immune cell composition in osteosarcoma

Utilizing CIBERSORT software, an assessment of the immune cell landscape within osteosarcoma (OS) tissue samples was performed. This evaluation aimed to understand the potential interaction between immune cell populations and the two apoptosis-linked genes, SERPINE2 and CPT1B, which are integral to the prognostic model.

**Figure 7:**
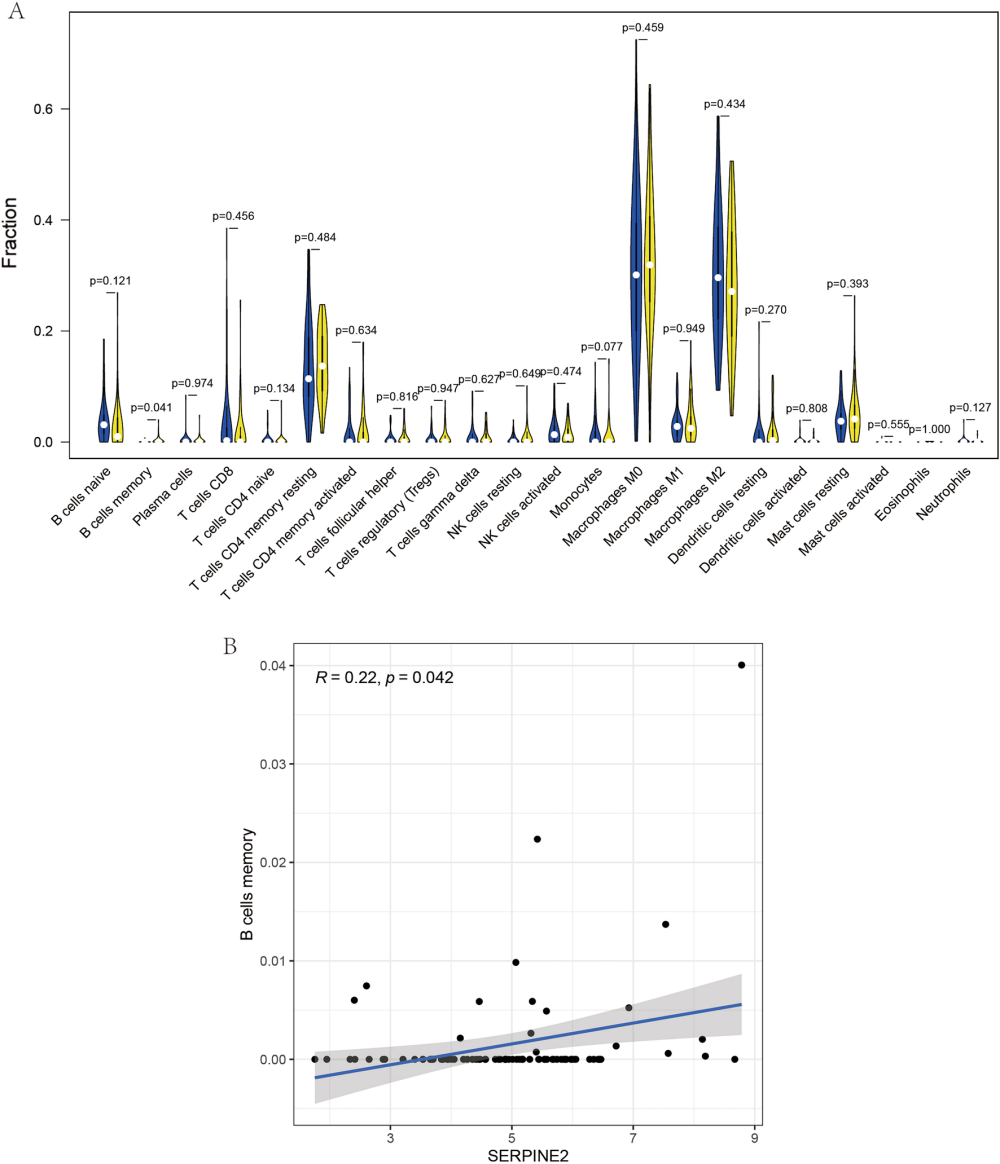
SERPINE2 and Immune Cell Correlation in OS. (**A**) This figure maps out the various immune cells associated with SERPINE2 within the context of osteosarcoma, indicating which types of immune cells show correlation with SERPINE2 expression levels. (**B**) This figure provides a correlation analysis graph between SERPINE2 and memory B cells specifically, detailing the strength and nature of the association, with data points representing individual samples that illustrate the trend of this relationship.

For SERPINE2, the analysis juxtaposed gene expression levels with the prevalence of different immune cells (Fig. 7A,B). The findings highlighted a notable positive correlation between the expression of SERPINE2 and the presence of memory B cells (R = 0.22, *p* < 0.05), suggesting an immunological interplay involving SERPINE2 in the context of OS.

**Figure 8:**
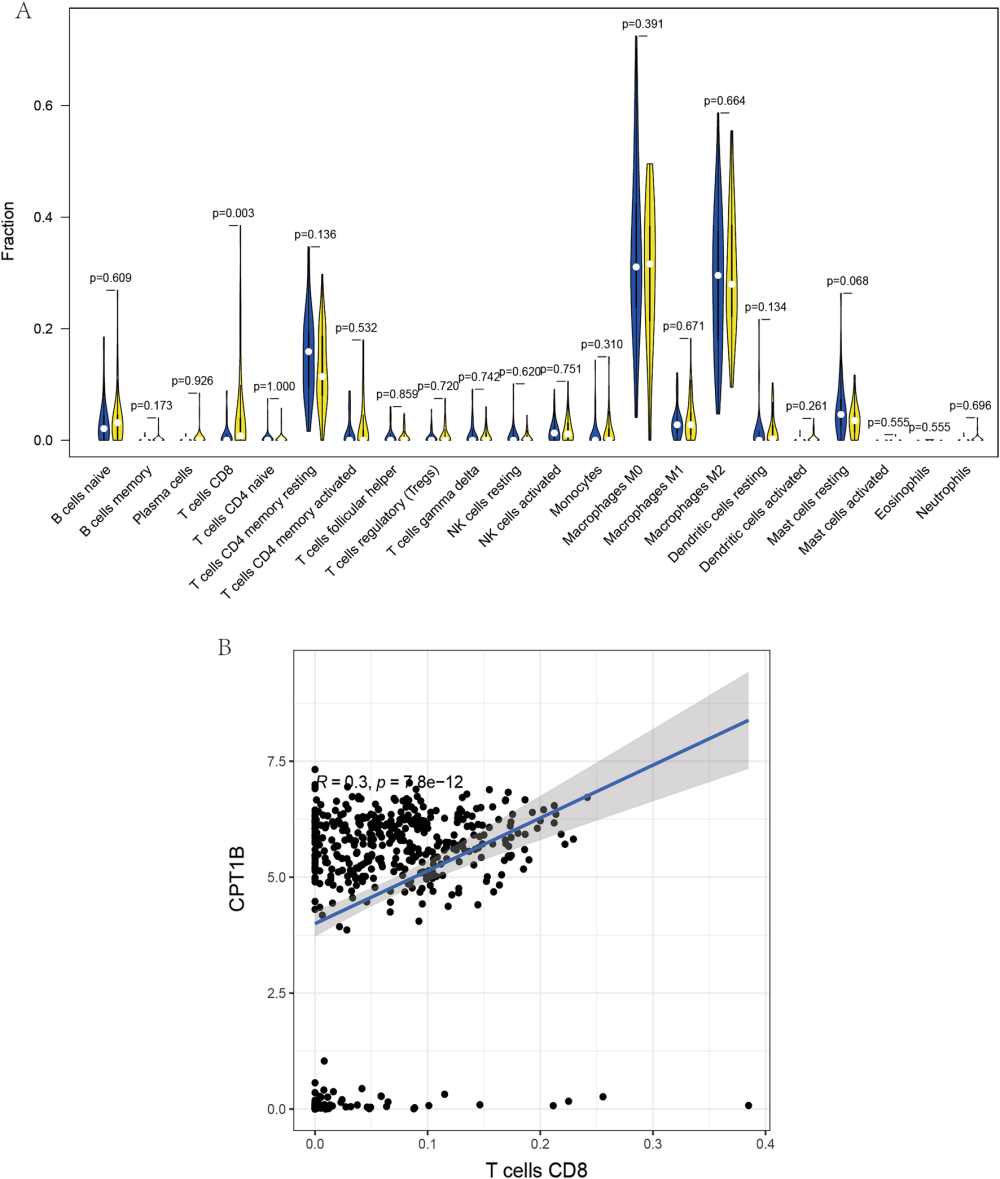
Correlation between CPT1B and Immune Cells in Osteosarcoma. (**A**) This illustration outlines the range of immune cells that demonstrate associations with the expression of CPT1B in osteosarcoma cases. It showcases the specific immune cell types whose activities may be linked to or affected by CPT1B levels. (**B**) This part of the figure presents a detailed correlation analysis focusing on the relationship between SERPINE2 expression and various immune cells. It graphically represents the correlation coefficients, with points on the graph indicating individual data points that highlight the pattern of interaction between SERPINE2 and the immune cells in the context of the disease.

Conversely, when assessing the relationship of CPT1B expression with immune cells (Fig. 8A,B), a substantial positive correlation emerged with CD8+T cells (R = 0.3, *p* < 0.001). This association underscores the relevance of CPT1B in the immune response within OS, particularly concerning cytotoxic T cell activity.

### Immunohistochemical validation in osteosarcoma tissues

**Figure 9:**
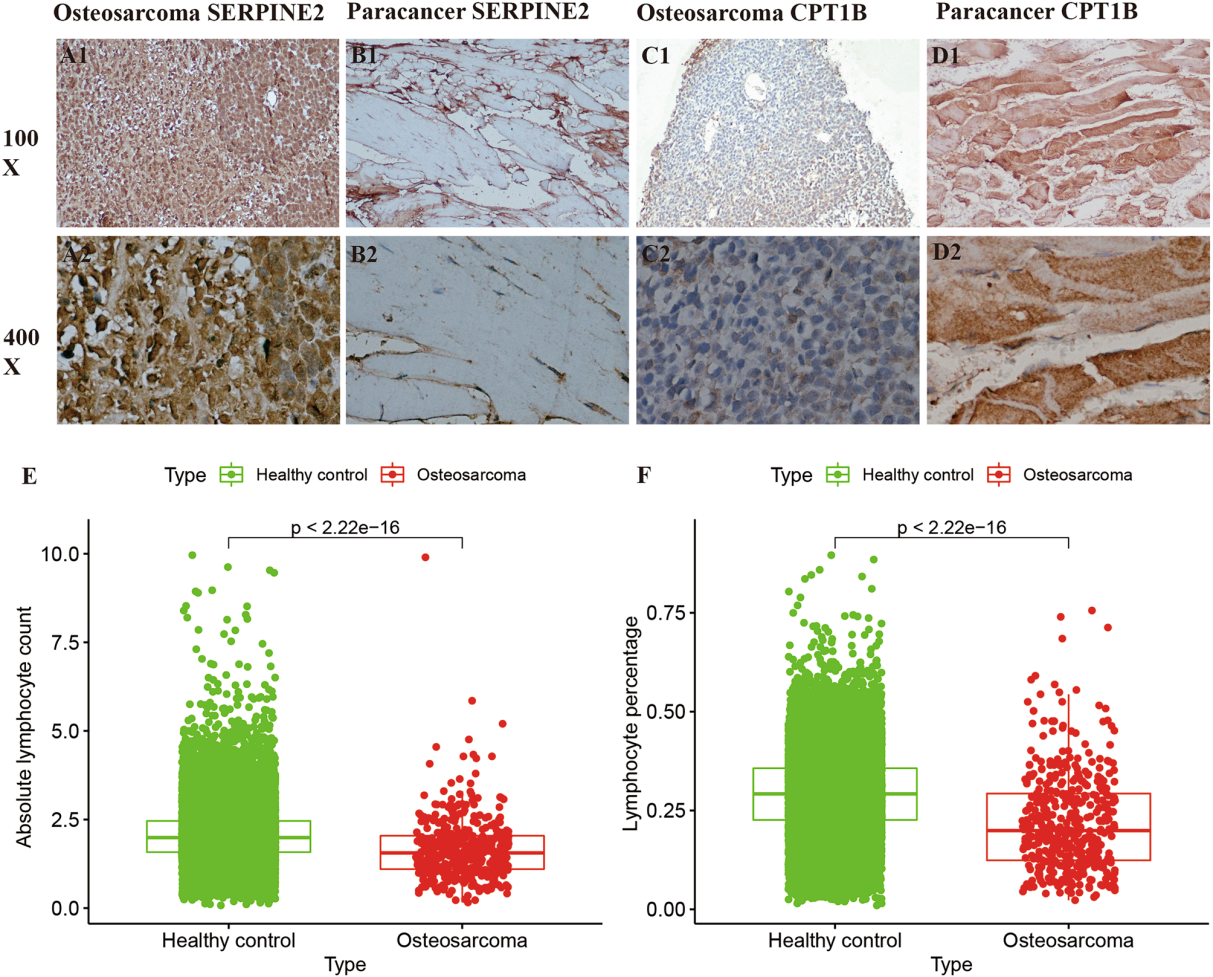
Comparative Analysis of Gene Expression and Routine Blood Parameters. (**A1**, **B2**) These panels display the results of immunohistochemical staining, contrasting the expression levels of SERPINE2 in osteosarcoma tissues versus adjacent non-cancerous tissues. A1 showcases staining in the osteosarcoma, and B2 depicts staining in the paraneoplastic tissues, highlighting the differential expression visually. (**C1**, **D2**) Similar to the above, these images exhibit the immunohistochemical analysis for CPT1B. C1 presents the expression in the osteosarcoma tissues, and D2 illustrates the expression in adjacent non-tumorous tissues, allowing for a side-by-side comparison of the gene expression levels in the pathological versus normal context. (**E**, **F**) These graphs outline the variations in routine blood analysis, specifically focusing on lymphocyte counts and percentages. Figure E represents the absolute lymphocyte counts and F shows the percentage of lymphocytes, both comparing data from healthy individuals against those with osteosarcoma, to provide insights into the systemic immune differences associated with the disease.

Pathological samples obtained from osteosarcoma (OS) surgeries at the First Clinical Affiliated Hospital of Guangxi Medical University were analyzed via immunohistochemistry to corroborate our bioinformatics findings. Figure 9A1–B2 display a marked elevation of SERPINE2 protein levels within OS tissues in comparison to adjacent non-tumorous tissues, as depicted by specific immunohistochemical staining intensity.

Contrarily, the CPT1B protein demonstrated a more pronounced presence in the surrounding non-tumorous tissues rather than in the OS tissues themselves (Fig. 9C1–D2), aligning with the gene expression trends identified in our bioinformatic analysis—SERPINE2 being upregulated in OS, whereas CPT1B showed downregulation. The immunohistochemical staining rates and their statistical significance are detailed in Table 3.

**Table 3:** Table 3 demonstrates the statistics of the positive rate of immunohistochemistry.

Gene name	Osteosarcoma positive area	Positive paracancer are
CPT1B	0.31	0.68
SERPINE2	0.72	0.22

Moreover, hematoxylin and eosin (H&E) staining provides additional insights; the OS cell nuclei are tightly packed, as opposed to the more dispersed arrangement seen in control tissue. Furthermore, a notable difference in staining intensity is observed—the nuclei of OS cells are darkly stained in contrast to the lighter staining of the control group nuclei (Fig. 9A1–B2), underscoring the histopathological differences between the neoplastic and non-neoplastic tissues.

### Comparative analysis of routine blood parameters in osteosarcoma

We scrutinized routine blood test data from 20,679 individuals diagnosed as free from osteosarcoma (OS) and other tumors, obtained between January 1, 2012, and January 1, 2022, from the First Affiliated Hospital of Guangxi Medical University. A cohort of 437 individuals diagnosed with OS was also evaluated.

**Figure 10:**
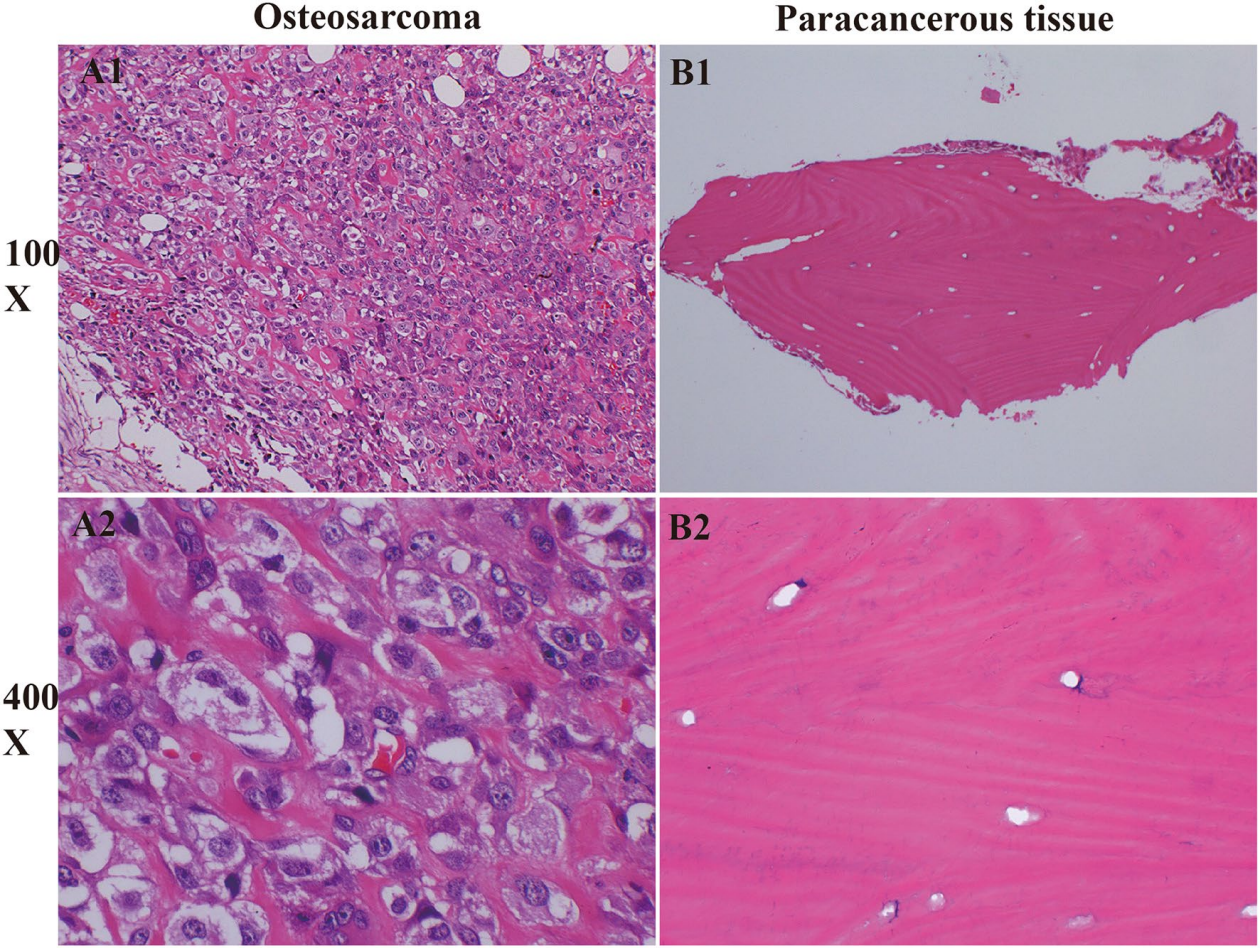
HE staining results picture. (**A1**, **A2**) These images depict the classic H&E staining of osteosarcoma (OS) tissues, as observed through an inverted microscope. A1 provides a broader view at a ×100 magnification, showing the general tissue architecture, while A2 offers a more detailed examination at ×400 magnification, highlighting cellular morphology and potential pathological features characteristic of OS. (**B1**, **B2**) In contrast, B1 and B2 present the H&E staining of control tissues, presumably healthy or non-tumorous tissue, under the same magnifications, ×100 and ×400, respectively. These images serve as a normative reference, allowing for direct visual comparison to identify differences in tissue structure, cell density, and organization between diseased and normal states.

**Figure 11:**
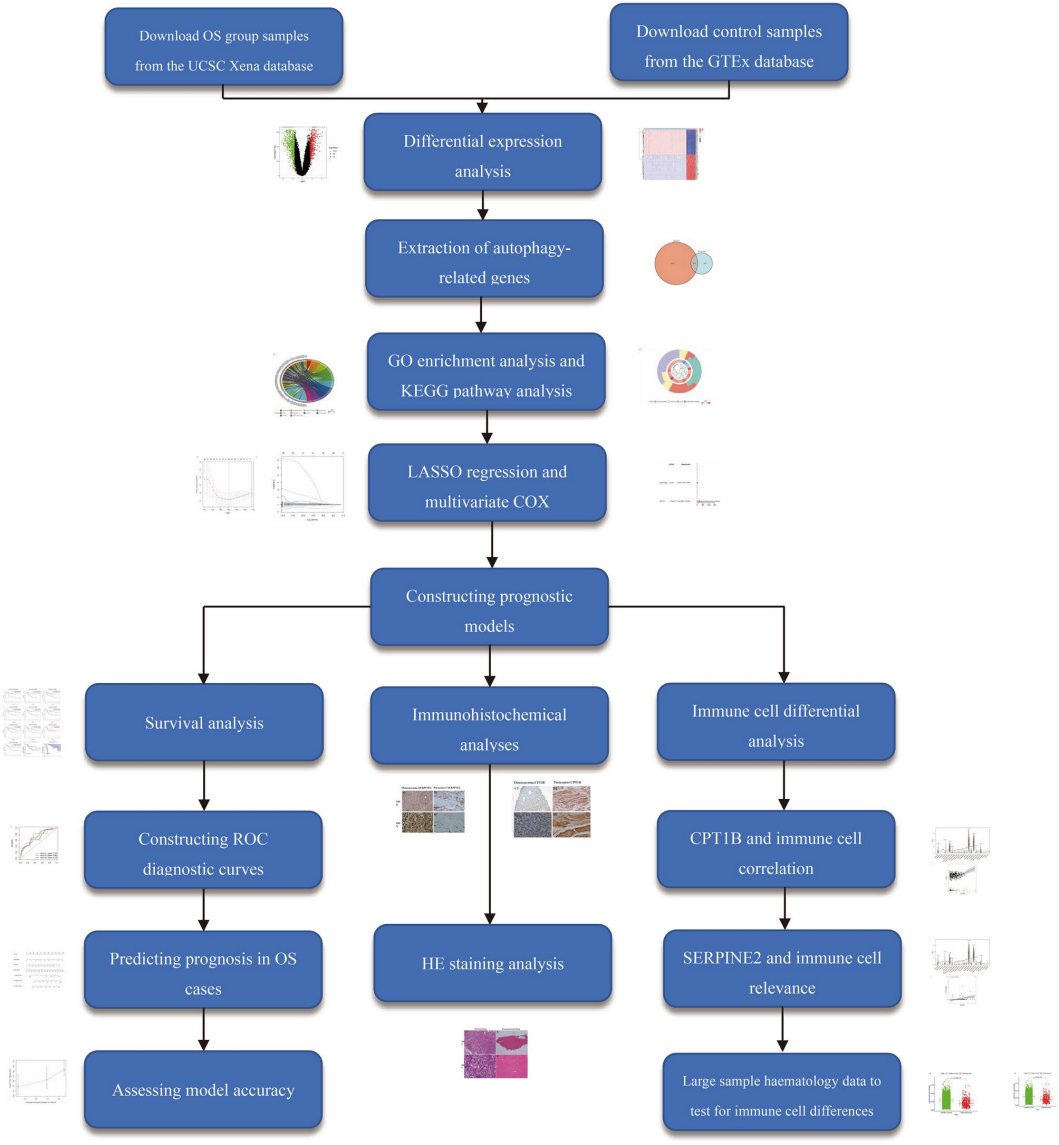
Work flow chart. Detailed flow chart of the work of this study.

Upon statistical examination of these datasets, we discerned a significant disparity in lymphocyte counts and percentages when comparing the healthy control group to the OS group, with the former exhibiting higher values (*p* < 0.001). These findings lend further credibility to our bioinformatics analysis, which indicated a decrement in lymphocyte levels in OS patients (Fig. 10E,F). This trend of lymphocyte diminution in OS could potentially serve as an additional hematological indicator for the disease state. See (Fig. 11) for the workflow diagram of this study.

## Discussion

In our analysis, GO and KEGG pathways were interrogated to understand the roles of apoptosis-associated differentially expressed genes in osteosarcoma (OS). GO terms were enriched in key cellular processes including collagen fibril and extracellular matrix organization, as well as nuclear and organelle division, indicative of their pivotal role in cellular integrity and replication. KEGG analysis highlighted the enrichment of these genes in pathways such as ECM-receptor interaction and cell cycle regulation, both imperative for tumor progression and metastasis. It has previously been reported that tumorigenesis and tumor progression, involved in the extracellular matrix (ECM), may also be involved in the development of colorectal cancer via interactions with other signaling pathways. This aligns with literature demonstrating ECM's involvement in colorectal cancer pathogenesis and the role of COL8A1 in breast cancer metastasis, independent of molecular subtype^19^. Moreover, our study corroborates emerging data on the immunomodulatory potential of CDK4/CDK6 inhibitors, beyond their well-documented cell cycle arrest capabilities in cancer therapy^20,21^. The apoptosis-related genes identified here show a strong association with tumorigenic processes, reinforcing the concept that apoptotic mechanisms are deeply intertwined with tumor development. These insights offer a novel reference for advancing the understanding of OS pathology and potentially, its therapeutic targeting.

The gene encoding Serpin Family E Member 2 (SERPINE2) has been implicated in disorders such as Visceral Heterotaxy and Ankylosing Spondylitis. Recent investigations have revealed a correlation between elevated SERPINE2 expression and reduced overall survival in patients, signifying its potential as a prognostic biomarker for lung cancer. This suggests that SERPINE2 may have broader implications in oncology, extending its relevance beyond the initially understood disease associations^22^. SERPINE2 has also been identified as a significant player in bladder cancer, where its expression levels are tightly linked with patient outcomes. Elevated SERPINE2 expression in bladder cancer is associated with a reduced overall survival rate, suggesting its utility as a predictive biomarker for patient prognosis in this malignancy. The implication of SERPINE2 in the pathophysiology of bladder cancer underscores its potential as a target for therapeutic strategies and diagnostic tools^23^. SERPINE2 has been found to have an integral role in papillary thyroid cancer, where its expression may affect tumor progression and patient prognosis^24^. Our investigation into osteosarcoma (OS) prognosis concerning apoptosis-related genes found that high SERPINE2 and CPT1B expression correlates with poor survival. Particularly, a prognostic model utilizing these genes classified patients into high and low-risk categories with significant survival disparities. Additionally, SERPINE2's association with immune cells was explored, revealing a positive correlation with memory B cells. This finding is in line with Helmink et al.'s research, which, through comprehensive single-cell RNA sequencing and flow cytometry, highlighted memory B cells' prevalence in tumors, suggesting their potential role in tumor immunity and possibly prognosis^25^. The present study aligns with the burgeoning evidence suggesting the critical role of memory B cells in the pathophysiology of osteosarcoma (OS). Scholarly research has substantiated immune cell imbalance as a central mechanism in oncogenesis^26–28^. Consistently, our investigation reveals a marked disequilibrium in immune cell populations within OS, positing this imbalance as a contributory element to the malignancy's progression.

The gene Carnitine Palmitoyltransferase 1B (CPT1B) encodes a protein that plays a pivotal role in lipid metabolism, with implications for conditions such as Visceral Steatosis and Carnitine Palmitoyltransferase I Deficiency. Research by Vantaku et al. has integrated metabolomic, lipidomic, and transcriptomic methodologies, unveiling a correlation between diminished CPT1B expression and the severity of tumor grade. Their findings suggest a parallel decrease in fatty acid oxidation (FAO) in high-grade bladder cancer and an associated reduction in acylcarnitine concentrations^29^. The upregulation of Carnitine Palmitoyltransferase 1B (CPT1B) has been identified as a prognostic marker in prostate cancer, with higher levels of expression linked to poorer patient outcomes. This suggests the potential utility of CPT1B as a biomarker for assessing prognosis in prostate cancer cases^30^. Moreover, the development of chemoresistance in tumor cells, which significantly diminishes the efficacy of chemotherapy, can result in adverse prognoses. Research indicates that the m6A modification-induced Estrogen-Related Receptor Gamma (ERRγ) might promote chemoresistance in malignant cells through the upregulation of CPT1B, thereby posing a challenge for effective chemotherapy^31^. The current findings align with this study, where an osteosarcoma (OS) prognostic model leveraging two genes, including CPT1B, predicted significantly poorer outcomes in the high-risk group versus the low-risk group. Furthermore, analysis of OS-associated immune cells highlighted a substantial positive link between CPT1B and CD8+T cells. Notably, CD8+T cells are implicated in the advancement of cancer, with reports suggesting that T cell dysfunction correlates with the compromised T cell activity frequently observed in human malignancies^32^. The current research corroborates the critical prognostic role of CD8+T cells in osteosarcoma (OS), as evidenced by the significant positive correlation between these immune cells and CPT1B expression. Notably, CPT1B expression is dysregulated and markedly elevated in OS. This suggests that the upregulation of CPT1B may drive the increased activity of CD8+T cells, contributing to OS progression through intricate mechanisms. These insights offer a novel reference for further OS research, underscoring the intricate interplay between immune modulation and tumor development.

In our study, the prognostic model identified certain genes, notably SERPINE2 and CPT1B, as having a strong correlation with the prognosis of osteosarcoma (OS). These discoveries highlight the potential therapeutic implications of these genes and their association with specific immune cell types. The key insights are as follows: First, the Therapeutic Potential of SERPINE2 and CPT1B: The expression levels of these genes indicate their potential as therapeutic targets. An abnormally high expression of SERPINE2 or CPT1B could prompt the development of small molecule inhibitors or monoclonal antibodies targeting these genes. Such a strategy might be effective in regulating the growth and apoptosis of tumor cells, potentially inhibiting tumor progression. Second, the Association with Immune Cells: Our research also revealed a positive correlation between the expression of SERPINE2 and CPT1B and the presence of specific types of immune cells. Specifically, the expression of SERPINE2 positively correlates with the presence of memory B cells, while CPT1B expression is positively associated with the presence of CD8+T cells. These findings suggest new possibilities for future immunotherapeutic strategies.

In this study, four AI-based methods were employed to identify differentially expressed genes in osteosarcoma (OS), leading to the creation of prognostic models through logistic regression. These models offer fresh insights and directions for predicting OS outcomes. Additionally, examining the link between these genes and immune cell populations revealed a notable positive correlation of SERPINE2 with memory B cells and CPT1B with CD8+T cells. Such findings suggest that the dysregulation of SERPINE2, CPT1B, and immune cell imbalance may contribute to the advancement of OS. Immunohistochemical staining validated the differential expression of these genes in OS versus adjacent non-tumor tissues, aiding in prognostic model development. Moreover, the analysis of routine blood data from an extensive cohort validated the immune cell differences, laying new groundwork for immunotherapy strategies in combating OS, a disease characterized by a high rate of metastasis.

Our investigation acknowledges several limitations, including a modest sample size, suboptimal utilization of clinical data, and insufficient laboratory validation of our analyses. These constraints highlight the need for further, more extensive research to substantiate our findings.

## Conclusion

Dysregulation of SERPINE2 and CPT1B, along with lymphocyte imbalances, emerges as a pivotal molecular pathway in the etiology of osteosarcoma (OS). These findings suggest that both SERPINE2 and CPT1B hold potential as prognostic biomarkers for OS.

## Data Availability

Data downloaded from the publicly available databases GTEx database (https://www.gtexportal.org/home/) and UCSC Xena database (http://xena.ucsc.edu/) and GEO database (https://www.ncbi.nlm.nih.gov/gds/). GSE21257(https://www.ncbi.nlm.nih.gov/geo/query/acc.cgi?acc=GSE21257).
